# Simulation of Ectopic Pacemakers in the Heart: Multiple Ectopic Beats Generated by Reentry inside Fibrotic Regions

**DOI:** 10.1155/2015/713058

**Published:** 2015-10-25

**Authors:** Bruno Gouvêa de Barros, Rodrigo Weber dos Santos, Marcelo Lobosco, Sergio Alonso

**Affiliations:** ^1^Graduate Program in Computational Modeling, Federal University of Juiz de Fora, 36036-330 Juiz de Fora, MG, Brazil; ^2^Department of Applied Physics, Universitat Politécnica de Catalunya-BarcelonaTech, Barcelona, Spain

## Abstract

The inclusion of nonconducting media, mimicking cardiac fibrosis, in two models of cardiac tissue produces the formation of ectopic beats. The fraction of nonconducting media in comparison with the fraction of healthy myocytes and the topological distribution of cells determines the probability of ectopic beat generation. First, a detailed subcellular microscopic model that accounts for the microstructure of the cardiac tissue is constructed and employed for the numerical simulation of action potential propagation. Next, an equivalent discrete model is implemented, which permits a faster integration of the equations. This discrete model is a simplified version of the microscopic model that maintains the distribution of connections between cells. Both models produce similar results when describing action potential propagation in homogeneous tissue; however, they slightly differ in the generation of ectopic beats in heterogeneous tissue. Nevertheless, both models present the generation of reentry inside fibrotic tissues. This kind of reentry restricted to microfibrosis regions can result in the formation of ectopic pacemakers, that is, regions that will generate a series of ectopic stimulus at a fast pacing rate. In turn, such activity has been related to trigger fibrillation in the atria and in the ventricles in clinical and animal studies.

## 1. Introduction

Cardiac arrhythmias are malfunctions of the coordinated dynamics of heart electric activity. In the extreme case when many cardiac myocytes are strongly desynchronized, contraction is impaired. This underlying complex spatiotemporal dynamics is called fibrillation and can take place in the atria or ventricles. Reentry that is the reappearance of the action potential wave after its first passage or spiral wave is believed to be a necessary but not sufficient condition for fibrillation. Once one or more spiral waves exist, they can interact or a single one can become unstable and in either situation this may lead to fibrillation. Spiral generation, spiral breakup, instabilities, and fibrillation have been extensively studied by the use of computational models [1–3], clinical data, or animal models [4–7]. One of the most common protocols to generate a spiral wave is via an ectopic beat: an additional stimulus generates a second wave that will interact with the sinus wave and lead to the genesis of a spiral wave or reentry. This process is well understood and many known situations lead to the appearance of an ectopic beat in pathological or physiological conditions. Just to name two of them, in a healthy heart, an external impact may stretch some region of the heart and via stretch-activated ion channels, cardiac myocytes may coordinately generate action potentials that may initiate a second wave [8]. In an infarct region, gap junctional remodeling, fibroblast to myocyte coupling, and fibrosis can significantly slow down wave propagation when it enters the infarct region, so that the wave leaves the region detached from the sinus wave, that is, when a second wave or ectopic beat appears [9].

There are many different protocols for the generation of fibrillation. Fast pacing is a common protocol used in both computational and animal models and experiments [5, 6, 10]. We can refer to a recent work [11] where atrial fibrillation was studied in an accurate model of human atria. In the mentioned work, it was only possible to induce atrial fibrillation by using a burst of at least six consecutive ectopic beats separated by 130 ms or via continuous fast periodic pacing (also with an ectopic beat period of 130 ms). Unfortunately, differently from the case of a single ectopic beat, there are not many well-understood examples or explanations for ectopic pacemakers that is a region that would generate multiple ectopic beats at a frequency much higher (near an order of magnitude) than the sinus rhythm. In particular, new evidences suggest that two mechanisms (combined or not) may play a very important role in the generation of ectopic pacemakers: abnormal intracellular calcium (Ca2+) signalling and microreentry (reentry inside a fibrotic region) [12].

In this work we were able to generate an ectopic pacemaker in computer simulations of cardiac electrophysiology by assuming a single hypothesis for substrate: the existence of a region with a mixture of excitable (healthy myocytes) and nonexcitable (fibrosis, for instance) areas, where each area has a minimum size of a single myocyte. This fibrotic region has been named before as microfibrosis [13–15], not mainly because of its whole size, but rather due to the microscopic separation of these two phases, myocytes and fibrosis, which as mentioned before is at the scale of a single myocyte.

The mechanism of this ectopic pacemaker is simple. The two different phases (excitable and nonexcitable) form a maze or labyrinth for wave propagation. Propagation becomes fractionated by the zig-zag like or tortuous pathways it has to follow. Macroscopic propagation is considerably slowed down. Microscopically, the topology of the maze allows the electric wave to reexcite the fibrotic tissue before the wave leaves it. Therefore, reentry and spiral waves are formed inside the fibrotic region. This spiral-like wave will continuously try to generate ectopic beats whenever it touches the border of the fibrotic region.

Evidences that support this thesis can be found in previous computational, animal, and clinical studies. Many animal studies have associated regions near fibrosis as triggers for ventricular tachycardia, fibrillation [9], or atrial fibrillation [16]. Macroscopically, it has been shown that propagation velocity in the border zone of infarct may drop from 0.51 m/s to 0.09 m/s [9]. Microscopically, propagation near fibrosis has been associated with fractionated electrograms, or complex fractionated atrial electrograms (CFAE), in both animal [17] and clinical studies [18]. These findings have remarkably changed the management and prognostics of patients with atrial fibrillation via a new procedure of catheter ablation of atrial fibrillation that is guided by CFAE mapping [19]. Recent clinical studies of guided CFAE ablation revisited the benefits and risks of this procedure and clearly suggest that further investigation on this topic is required [20–22].

Computer simulations gave valuable insights into how the microstructure of cardiac tissue, specifically microfibrosis, and CFAEs are related [13, 15]. These previous studies have used realist electrophysiology single myocyte models and detailed microscopic description to quantitatively represent the fine structure of cardiac tissue; that is, the models were based on subcellular discretizations of cardiac tissue.

However, up to now, a clear link between the microstructure remodeling of cardiac tissue (microfibrosis) and a theoretical substrate for cardiac arrhythmia (fibrillation) is still missing. Evidences of the importance of tissue heterogeneity, specifically of nonconductive cells, on spiral genesis and breakup were already presented in a theoretical study using simple models based on cellular automata [23]. Recently, Alonso and Bär [24] have presented, for the first time, simulations based on simple discrete and isotropic models of cardiac tissue that could generate ectopic pacemakers. The mechanism was the one explained before: reentry was generated inside a heterogeneous tissue (composed of active or nonactive discrete cells). In addition, Alonso and Bär [24] have described how the probability of reentry depends on a specific feature of the topology of the discrete network that was used to model cardiac tissue: the percentage of nonactive cells* phi*.

**Figure 1:**
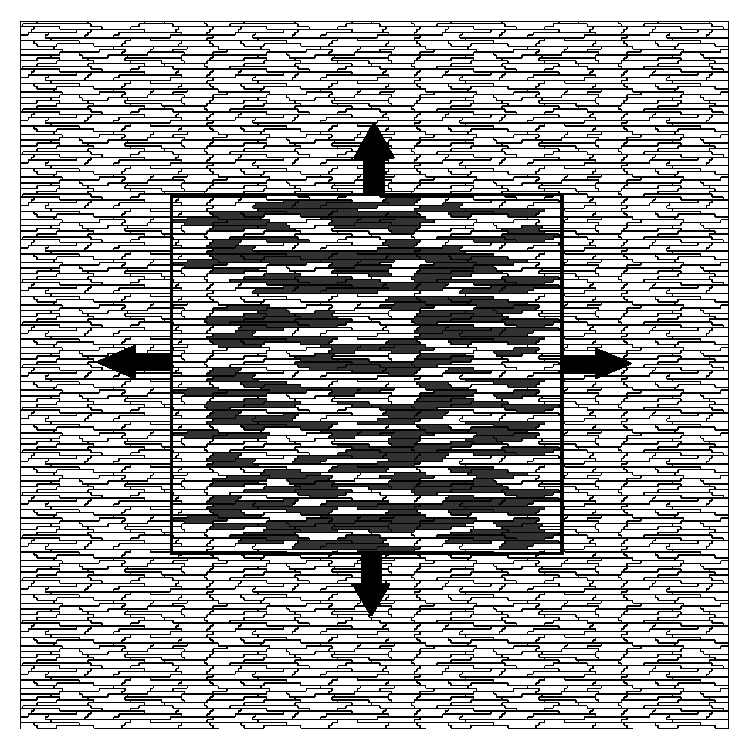
In the center of this figure we present the fibrotic region that is simulated in this work. The presence of reentry inside the fibrotic region may constantly reexcite the surrounding tissue and therefore act as an ectopic pacemaker.

The focus of this work is on bridging the gap between the quantitative and more complex models used to simulate microfibrosis and CFAEs [13] and the qualitative and simplified models used to relate microfibrosis and ectopic pacemaker [24]. For this purpose two different models were used, and both are as complex and realistic as those used before in [13]; that is, both use realistic description of single myocyte electrophysiology and account for the microstructure of cardiac tissue such as gap junction distribution and tissue anisotropy: (1) a microscopic model with subcellular discretization of 8 *μ*m as described before in [25] and (2) a discrete equivalent model that, to the best of our knowledge, has never been presented before. It is important to highlight that in this paper our models focus on the electric dynamics inside the ectopic pacemaker or microfibrosis region (see Figure 1). In the near future we will study how this activity propagates to the outside of this fibrotic region and ultimately influence the surrounding (nonfibrotic) regions of the heart.

Our simulation results support the thesis that the microstructure of a fibrotic tissue may indeed generate* per se* an ectopic pacemaker via reentry inside the microfibrosis. Also, as in [24], the probability of a fibrotic region to become an ectopic pacemaker depends on the percentage of fibrosis in the tissue. Using our realistic models we could quantify that ectopic pacemakers were easier to be generated with *ϕ* between 42% and 50%. Our results also gave new insights and suggest that, in addition to the percentage of fibrosis, *ϕ*, the probability of a fibrotic region to become an ectopic pacemaker also depends on the direction of the approaching plane wave. This is in agreement with the recent results presented in [15] where it was shown that the waveforms of CFAEs depend on both the structure or topology of the fibrosis and the direction of the approaching propagating wave.

## 2. Methods

### 2.1. A Detailed Microscopic Model of Mouse Ventricular Tissue

#### 2.1.1. Modeling Cardiac Microstructure

**Figure 2:**
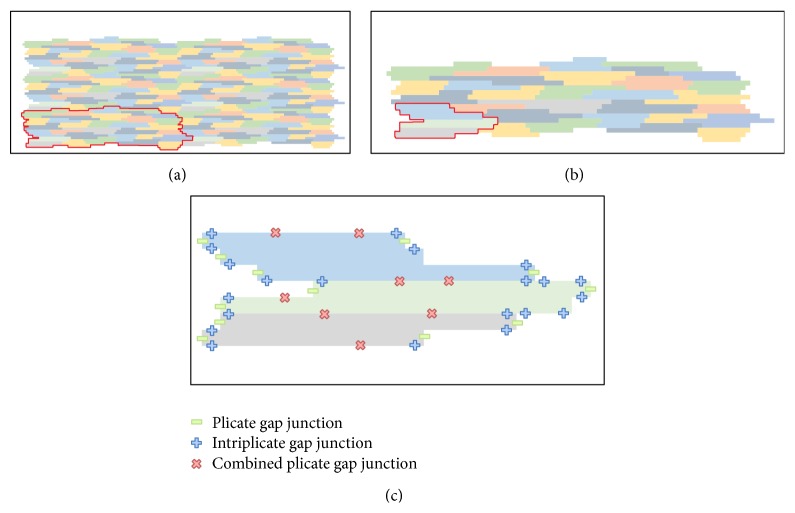
(a) Six basic units being combined to form a larger tissue. (b) Basic unit of cardiac myocyte distribution based on a total of 32 cells. (c) This figure presents an example of how different gap junctions are distributed in three neighboring myocytes that belong to the basic unit. There are only 5 possible types of connections between neighboring volumes that are membrane, which indicated no-flux between neighboring volumes; cytoplasm, which indicates the neighboring volumes are within the same cell; three possible types of gap junctions, plicate, intriplicate, and combined plicate.

We developed a two-dimensional model that is based on the previous work of Spach and collaborators [26, 27] that accounts for the microstructure of cardiac tissue, gap junction heterogeneous distribution, and discretizations of 8 *μ*m × 8 *μ*m. A basic template for myocyte connections was developed and is presented in Figure 2(b). This basic unit accounts for the connection of a total of 32 cardiac myocytes with different shapes and numbers of neighboring cells.

In the previous model of Spach and collaborators [26, 27] the basic template consisted of 33 myocytes with a discretization of 10 *μ*m × 10 *μ*m whereas in our template we have 32 myocytes with a discretization of 8 *μ*m × 8 *μ*m. These small modifications to the original model proposed in [26, 27] were adopted to better describe the values for cell length and width reported in the recent literature. The mean and SD (standard deviation) values for cell length and width are 120.9 ± 27.8 *μ*m and 18.3 ± 3.5 *μ*m, respectively. These values are close to those reported in the literature: [28] (length = 140 *μ*m and width = 19 *μ*m), [29] (length = 134 *μ*m and width = 18 *μ*m), and [30] (length = 100 *μ*m and width = 17.32 *μ*m). On average each cell connects to the other 6 neighboring myocytes. Our two-dimensional model considers a homogeneous depth *d* = 10 *μ*m [26, 27].

This basic unit was created in such a way that it allows the generation of larger tissue preparations via the connections of multiples instances of it.  Figure 2(a) presents how this can be achieved.

Figure 2(c) presents an example of how the connections between different myocytes can be arranged. The code was developed in a flexible way, so that it allows the user to set up for each discretized volume Vol_*i*,*j*_ (with area = *h*×*h*) conductivity or conductance values for the north (*σ*_*x*_*i*,*j*+1/2__), south (*σ*_*x*_*i*,*j*−1/2__), west (*σ*_*x*_*i*−1/2,*j*__), and east (*σ*_*x*_*i*+1/2,*j*__) volume faces. These can be any nonnegative values. In this work, we set the discretization *h* to 8 *μ*m. In addition, based on the work of Spach and collaborators [26, 27], we chose only 5 possible types of connections between neighboring volumes that are membrane (*σ*_*m*_ = 0.0), cytoplasm (*σ*_*c*_ = 0.4 *μ*S/*μ*m), gap junction plicate (*G*_*p*_ = 0.5 *μ*S), intriplicate (*G*_*i*_ = 0.33 *μ*S), and combined plicate (*G*_*c*_ = 0.062 *μ*S), where we use *σ* for conductivity and *G* for conductance. For the simulations presented in this work, the distribution of the different gap junctions within the 32 myocytes was not randomly generated. Instead, the gap junction distribution of the basic template unit was manually chosen to reproduce the distribution presented before in [26, 27]. With this setup and conductivity values we found that conduction velocity along the fibers was around 410 *μ*m/ms (LP) and was 130 *μ*m/ms transversal to fiber direction (TP). This results in a ratio LP/TP of 0.32, which is close to the conduction ratio reported in [26]. It is worth mentioning that the values of longitudinal and transversal conduction velocities observed in experiments vary significantly (longitudinal conduction velocity is between 30 and 70 cm/s under normal or control conditions). Indeed, this large variation of measurements are due to many factors, such as the experimental technique used, temperature, pacing protocol, species, and heart region. Our value for LP conduction velocity (41 cm/s) is within the range of those reported in the literature: rat left atria [31] (42 ± 17 cm/s), dog left ventricle [32] (63 ± 10 cm/s), mouse left and right ventricle [33] (35 ± 3 cm/s), and guinea pig left and right ventricle [34] (52 ± 2 cm/s).

#### 2.1.2. The Heterogeneous Monodomain Model

Action potentials propagate through the cardiac tissue because the intracellular space of cardiac cells is electrically coupled by gap junctions. In this work, we do not consider the effects of the extracellular matrix. Therefore, the phenomenon can be described mathematically by a reaction-diffusion type partial differential equation (PDE) called monodomain model, given by

(1) βCm∂Vx,y,t∂t+βIionVx,y,t,ηx,y,t=∇·σx,y∇Vx,y,t+Istimx,y,t,∂ηx,y,t∂t=fVx,y,t,ηx,y,t,

where *V* is the variable of interest and represents the transmembrane voltage, that is, the difference between intracellular to extracellular potential; **η** is a vector of state variables, which also influence the generation and propagation of the electric wave, and usually includes the intracellular concentration of different ions (K^+^, Na^+^, Ca^2+^) and the permeability of different membrane ion channels; *β* is the surface-volume ratio of heart cells; *C*_*m*_ is the membrane capacitance; *I*_ion_ the total ionic current, which is a function of *V* and a vector of state variables **η**; *I*_stim_ is the current due to an external stimulus; **σ** is the monodomain conductivity tensor. We assume that the boundary of the tissue is isolated; that is, no-flux boundary conditions were imposed (**n** · *σ*∇*V* = 0 on ∂*Ω*).

In this work, the Bondarenko et al. model [35] that describes the electrical activity of left ventricular cells of mice was considered to simulate the kinetics of *I*_ion_ in (1). The Bondarenko et al. model (BDK) was the first model presented for mouse ventricular myocytes [35]. The ionic current term *I*_ion_ in this model consists of the sum of 15 transmembrane currents. In short, Bondarenko's model is based on an ordinary differential equation (ODE) with 41 differential variables that control ionic currents and cellular homeostasis. In this model most of the ion channels are represented by Markov chains (MCs).

#### 2.1.3. Numerical Discretization in Space and Time

The finite volume method (FVM) is a mathematical method used to obtain a discrete version of partial differential equations. This method is suitable for numerical simulations of various types of conservation laws (elliptical, parabolic, or hyperbolic) [36]. Like the finite element method (FEM), the FVM can be used in several types of geometry, using structured or unstructured meshes and generates robust numerical schemes.

The development of the method is intrinsically linked to the concept of flow between regions or adjacent volumes; that is, it is based on the numerical calculation of net fluxes into or out of a control volume. For some isotropic problems discretized with regular spatial meshes, the discretization obtained with the FVM is very similar to the one obtained with the standard finite difference method (FDM). Detailed information about the FVM applied to the solution of monodomain can be found in [37, 38].

The reaction and diffusion parts of the monodomain equations were split by employing the Godunov operator splitting [39]. Therefore, each time step involves the solution of two different problems: a nonlinear system of ODEs

(2) ∂V∂t=1Cm−IionV,η+Istim,∂η∂t=fV,η;

and a parabolic PDE

(3) $$\begin{array}{c} \beta \left(\;C_{m}\frac{\partial V}{\partial t}\;\right)=\nabla \cdot \left(\;\sigma \nabla V\;\right). \end{array}$$

For the discretization of the nonlinear system of ODEs we note that its stiffness demands very small time steps. For simple models based on Hodgkin-Huxley formulation this problem is normally overcome by using the Rush-Larsen (RL) method [40]. However, for the most modern and complex models that are highly based on MCs, the RL method seems to be ineffective in terms of allowing larger time steps during the numerical integration. For the case of the Bondarenko et al. model, we tested both methods, Euler and RL, and both demanded the same time step, Δ*t*_*o*_ = 0.0001 ms due to stability issues. Since the RL method is more expensive per time step than the Euler method, in this work, we used the simple explicit Euler method for the discretization of the nonlinear ODEs.

Since the spatial discretization of our model, *h*, is extremely small, the CFL [41] condition that assures numerical stability is very restrictive. Therefore, for the PDE we used the unconditionally stable implicit Euler scheme. In addition this allowed us to use a longer time step for the numerical solution of the PDE (Δ*t*_*p*_ > Δ*t*_*o*_). The time derivative presented in (3), which operates on *V*, is approximated by a first order implicit Euler scheme.

The diffusion term in (3) must be discretized in space. For the space discretization we considered a two-dimensional uniform mesh, consisting of regular quadrilaterals (called “volumes”). Located in the center of each volume is a node. The quantity of interest *V*_*i*,*j*_ is associated with each node of the mesh (*ih*, *jh*), where *h* is the spatial discretization.

After defining the mesh geometry and dividing the domain in control volumes, the specific equations of the FVM can be presented. Equation (3) can be integrated spatially over an individual volume Vol_*i*,*j*_ of size *h* ^2^ *d*. Substituting the discretizations of the equations and decomposing the operators as described by (2) and (3) yield 
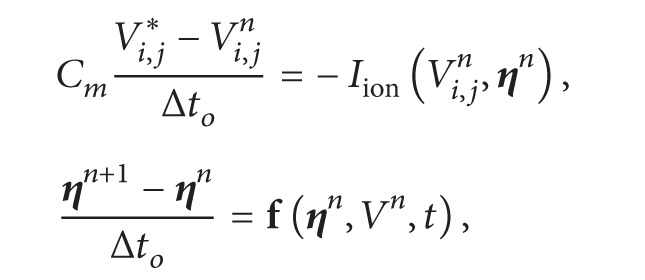
(4)

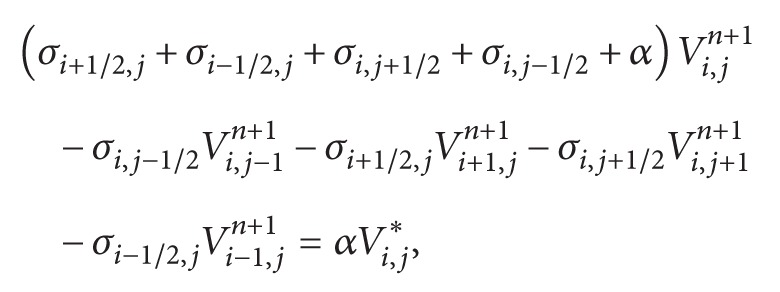
(5)where *α* = (*βC*_*m*_ *h* ^2^)/Δ*t*_*p*_, *n* is the current step, *∗* is an intermediate step, and *n* + 1 is the next time step. In addition *σ* can stand for any of the gap junction conductance (*G*_*p*_, *G*_*i*_, *G*_*c*_) divided by the depth *d* or for any conductivity value (*σ*_*c*_, *σ*_*m*_) defined for each volume face as described in Section 2.1.1. This defines the equations for each finite volume Vol_*i*,*j*_. First we solve the nonlinear system of ODEs associated with (4) *N*_*o*_ times until we have *N*_*o*_Δ*t*_*o*_ = Δ*t*_*p*_; then we solve the linear system associated with (5) to advance time by Δ*t*_*p*_.

### 2.2. An Equivalent Discrete Model of Mouse Ventricular Tissue

#### 2.2.1. A Discrete Model

Recent studies on discrete or discontinuous nature of propagation of the action potential have avoided the computational challenges that arise from microscopic models, through the development and use of discrete models, where each cardiac myocyte is represented by a single point (cell) contact with neighboring myocytes via discrete conductivities [24, 42]. This description has enabled the study of the effects that random distributions of conductivities have on conduction velocity and on the formation of patterns of reentry in cardiac tissue.

Discrete models were introduced by Keener in [43] to describe the electrical propagation in a 1D cable with nc cells connected to the case of low coupling of gap junctions. In this model, cells are considered isopotentials. Recently, in a work of Costa and Dos Santos [44], a comparison between discrete and microscopic models of a 1D cable of cells was presented.

In this work, an equivalent discrete model to the 2D microscopic model presented in Section 2.1 was developed. For this, using the cardiac microstructure presented in Section 2.1 and identifying the neighbors (links) of each cell, the conductance between each link was calculated and a new equivalent discrete model was generated.

**Figure 3:**
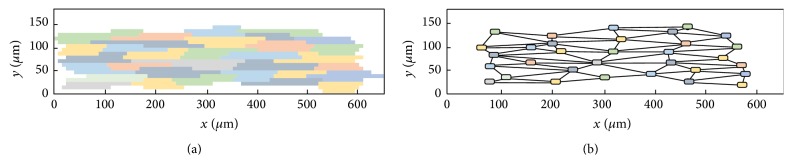
Elementary tissue unit in the microscopic (a) and the discrete (b) model. The same color code of each cell is employed in both meshes. Lines in the discrete model represent the connection between the linked cells.

In the microscopic model we had a uniform two-dimensional mesh consisting of regular quadrilaterals (called volumes). For the discrete model, each myocyte is represented by a single node or cell of a network, and each cell has different geometry (Vol_*i*_ = *A*_*i*_ *∗d*) and a different number of neighboring cells (nn_*i*_).  Figure 3 shows the resulting discrete topology for a basic unit of our microscopic mesh.

The equations that govern the propagation of action potentials in the cardiac tissue are similar to those used in the microscopic model presented in Section 2.1:

(6) VoliβCm∂Vi∂t+IionVi,ηi=∑j=1nniGi,j∗Vj−Vi+Iistim,∂ηi∂t=fVi,ηi,

for each node *i* of the discrete topology, where *G*_*i*,*j*_ is the equivalent conductance between cell *i* and cell *j*.

The time discretization is done in the same way as in the microscopic model (Section 2.1). As before, the reaction and diffusion parts of the monodomain equations were split by employing the Godunov operator splitting [39]. Therefore, each time step involves the solution of two different problems: a nonlinear system of ODEs and a coupled linear system for the discrete diffusion equation:
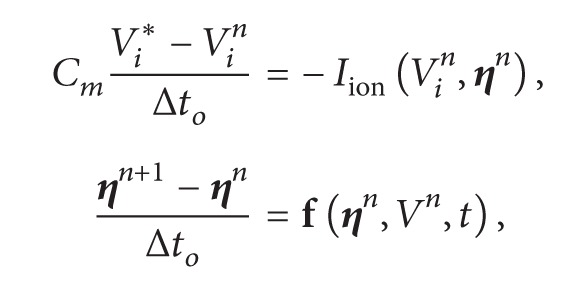
(7)

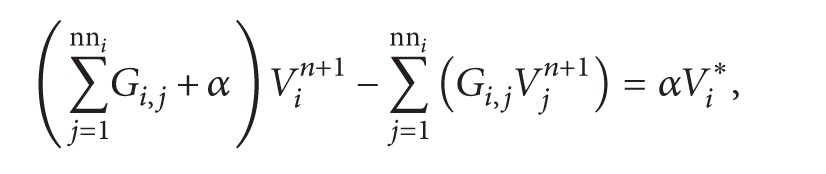
(8)where *α* = (*βC*_*m*_ *A*_*i*_ *d*)/Δ*t*_*p*_, *n* is the current step, *∗* is an intermediate step, and *n* + 1 is the next time step. This defines the equations for each cell *i*.

#### 2.2.2. Calculation of the Conductance between Neighboring Cells

To close the description of the discrete model presented in the previous section, we need to define the conductance values of the connections; that is, we need the values for *G*_*i*,*j*_ in (8).

This is done by applying the concept of combination of resistors in parallel to the microstructure defined in Section 2.1.1 for each pair of neighboring cells. From the association of resistors we have

(9) $$\begin{array}{c} \frac{1}{R_{i,j}}=\overset{\text{ng}}{\underset{k=1}{\sum }}\left(\;\frac{1}{R_{i,j,k}}\;\right), \end{array}$$

where *R*_*i*,*j*_ is the equivalent resistance and ng is the amount of gap junctions that connects cell *i* to cell *j*. The term *R*_*i*,*j*,*k*_ is calculated as a combination of resistors in series:

(10) $$\begin{array}{c} R_{i,j,k}={\mathrm{dist}}_{i,j,k}*\frac{1}{\left(\sigma h\right)}+\frac{1}{G_{k}}, \end{array}$$

where dist_*i*,*j*,*k*_ is the amount of volumes connected by cytoplasm that the current need to cross to go from cell *i* to cell *j* passing through gap junction *k*, which is calculated by the equation:

(11) dist⁡i,j,k=pri,x−gpi,k,x+pri,y−gpi,k,y+prj,x−gpj,k,x+prj,y−gpj,k,y,

where pr_*i*_ is a two-dimensional coordinate that represents the volume of the reference point of the cell *i* and gp_*i*,*k*_ is a two-dimensional coordinate that represents the volume that contains the gap junction connection *k* in cell *i*. It should be noted that these coordinates are index integers of the volumes in the mesh, so they do not have unit length. Thus each volume connected by the cytoplasm or gap junction represents a resistor.

In a simple way the term dist*∗*(1/(*σh*)) represents the total cytoplasmic resistance that the current faces to go from cell *i* to cell *j* or vice versa. The term 1/*G*_*k*_ represents the resistance of the gap junction. The conductivity values cytoplasm *σ* and conductance *G* for each type of gap junction are the same as defined in Section 2.1.1.

Thus we can now calculate the conductance *G*_*i*,*j*_ that is the inverse of the resistance:

(12) $$\begin{array}{c} G_{i,j}=\frac{1}{R_{i,j}}. \end{array}$$

### 2.3. Modeling Thousands of Fibrotic Regions

The sophisticated method presented in [15] to model fibrosis involves a huge pipeline based on histology acquisition, image processing, segmentation, and mesh generation. The purpose of our work is to assess the probability of a certain fibrotic tissue to become an ectopic pacemaker and to study how this depends on the fraction of fibrosis, *ϕ*. To achieve this goal it is necessary to perform thousands of simulations of different microfibrosis models. Therefore, the method presented in [15] does not fit to our purpose. We could, however, proceed with our study by using a similar method as presented in [13].

**Figure 4:**
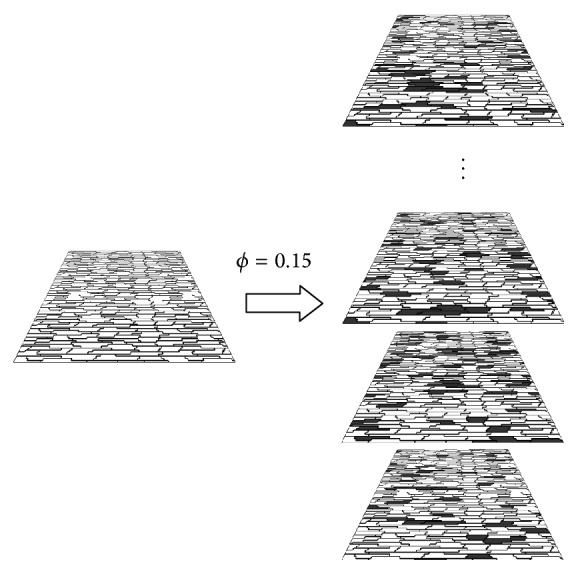
Process for the generation of one hundred of different tissue models with the same fraction, *ϕ*, of fibrosis.

For a given value of *ϕ*, we randomly generate a microfibrosis tissue by transforming some of the myocytes of our model in fibrosis, that is, nonconducting material. This was performed by removing all the gap junctions of the chosen myocytes, so that any propagation into or out of this myocyte is blocked. One hundred of realizations of different microfibrosis but with the same percentage of nonactive cells, *ϕ*, were simulated. This process is represented by Figure 4. The value of *ϕ* was varied from 40% to 55%, in steps of 1%. After each microfibrosis model is created using the microscopic model the equivalent discrete model is generated following the procedure explained in Section 2.2. For all the results presented in the next section the modeled cardiac tissues had dimensions of 1 cm × 1 cm.

## 3. Results

We employ numerical simulations to compare the two models described in Section 2 under two different situations: completely homogeneous distribution of healthy cells and the randomly inclusion of nonconducting cells. First we compare the dynamics of wave propagation and functional reentry in both models under homogeneous conditions. Then, we employ both models to study the problem of anatomical reentry in heterogeneous cardiac tissue.

The main advantage of the discrete model is the fast execution times when compared to those of the microscopic model. As we will present in the next sections this will be of extreme importance since we will present the simulations of over three thousands of different microfibrosis models.  Table 1 compares the execution times of the parallel implementations of the microscopic versus the discrete models. The simulations were executed on a parallel environment composed of 64 CPU cores and 16 GPUs as previously described in [25]. The parallel implementation and environment were able to reduce the execution time of the microscopic model from more than 6 days to 22 minutes. In addition, the parallel implementation of the discrete model takes only near one minute for the execution of the same simulation.

### 3.1. Wave Propagation in Homogeneous Models of Cardiac Tissue

In order to compare both models, we perform numerical simulations under homogeneous conditions and compare the spatiotemporal dynamics of the action potential waves.

#### 3.1.1. Traveling Waves in Homogeneous Models

**Figure 5:**
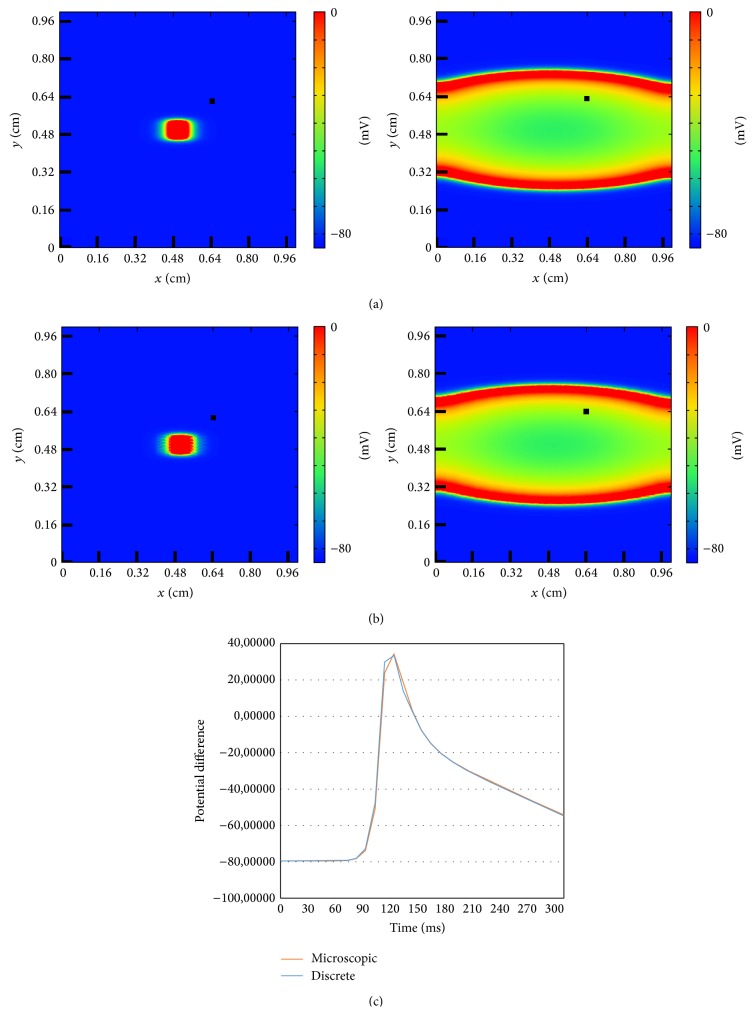
Comparison of the dynamics of a traveling wave of action potential generated by an initial centered perturbation in the discrete and in the microscopic models. (a) Evolution of the wave in the discrete model for *t* = 10 ms and *t* = 190 ms. (b) Evolution of the wave in the microscopic model for *t* = 10 ms and *t* = 190 ms. (c) Comparison of the time evolution of a single point between the wave in the discrete model and the wave in the microscopic model. The point is marked in the snapshots with a black square.

The two models described in the previous section gives rise to equivalent waves of action potential under the same conditions. For a quantitative comparison see Figure 5. An initial centered perturbation produces a concentric wave which propagates to the border of the medium. Its elliptic shape is due to the anisotropy of the tissue, which allows the wave to propagate faster in the longitudinal direction (to the left or to the right, see Figure 5) than in the vertical direction (bottom-up). The good agreement in the qualitative comparison between the snapshots at the same time shown at the top of Figure 5 is confirmed by the quantitative measurement of the action potential in a single cell of the tissue; see bottom of Figure 5. Under homogeneous conditions both models produce quantitatively the same types of waves because the connectivities among cells are equivalent (see Section 2.2.2) and, therefore, velocity of propagation is the same.

#### 3.1.2. Functional Reentry in Homogeneous Models

**Figure 6:**
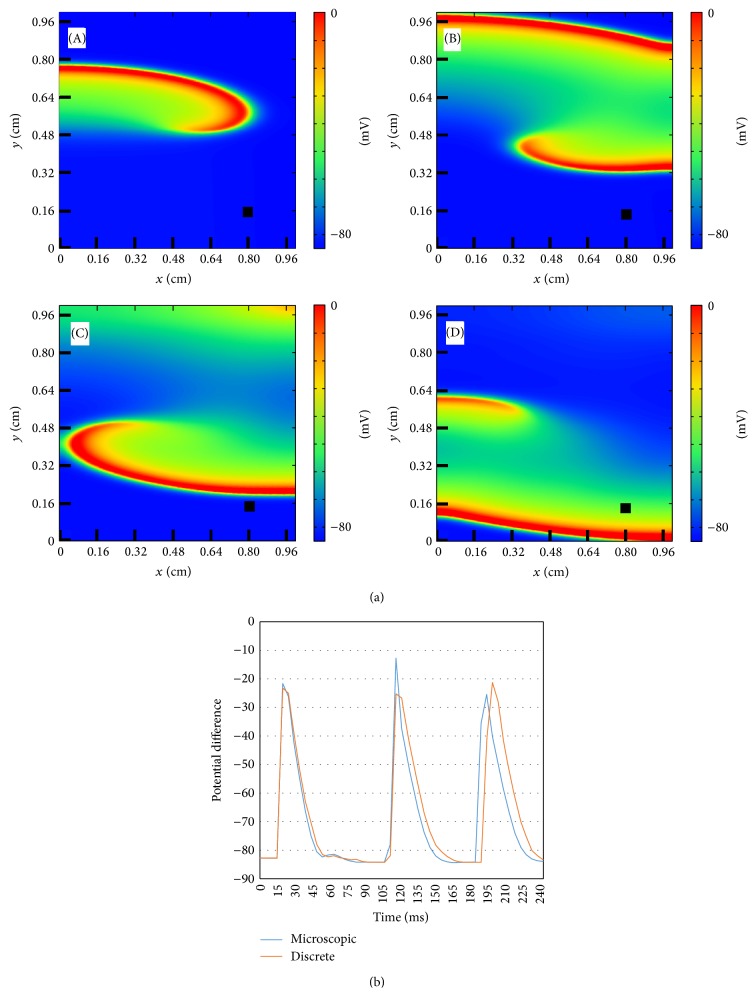
Dynamics of the transmembrane potential showing a spiral wave generated by a S1-S2 protocol using the microscopic model. A sustained, single, and stable spiral wave is generated using a S1-S2 protocol. (a) Four snapshots showing the spiral rotation corresponding to times: *t* = 60 ms (A), *t* = 80 ms (B), *t* = 110 ms (C), and *t* = 120 ms (D). (b) Comparison of the time evolution of AP at a single point between the microscopic model (see previous panel) and the discrete model (not shown). The point is marked in the snapshots with a black square.

The activity of the membrane action potential wave can remain in the tissue if there is a reentry. This can be produced by the S1-S2 protocol which consists in the reexcitation of the tissue (ectopic beat) after the pass of a normal traveling wave [25]. In Figure 6 an example of rotating spiral wave of action potential in the microscopic model of the tissue is shown. There is a repeated reexcitation of the tissue due to the reentry of the spiral wave and its rotation around a free end.

The action potential measured in a single cell (marked with a black square in the snapshots of the simulations) is shown in the bottom part of Figure 6 and compared with the equivalent simulation with the discrete model. The agreement is remarkable taking into account the different levels of approximation used by the two models. Thus, complex dynamics is well captured by the discrete model in comparison with the microscopic model.

### 3.2. Wave Propagation in Heterogeneous Models of Cardiac Tissue

Further comparisons between both models for heterogeneous conditions at the tissue level are shown in the next sections. Following, we remove a fraction of cells; that is, we randomly choose some cells which are then completely isolated from the rest of the tissue and cannot be excited anymore. Such cells represent the effect of the different types of heterogeneities in the tissue: damaged cells, damaged connections, fibrosis, extracellular matrix, or dead cells. We define as control parameter the fraction of isolated cells *ϕ*; that is, *ϕ* = 0 corresponds to the homogeneous limit discussed in the previous section and *ϕ* = 1 to a completely inert piece of tissue.

#### 3.2.1. Anatomical Reentry in the Discrete Model

**Figure 7:**
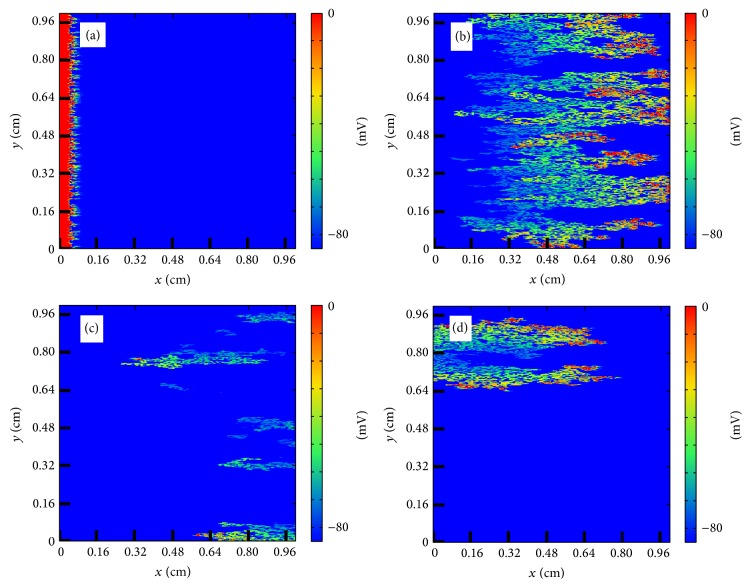
Reentry of action potential generated by the heterogeneous structure of the tissue in the discrete model. (a) Generation of the traveling wave in the left part of the tissue (*t* = 2 ms). (b) Wave rapidly propagates to the right following the direction of the fibers (*t* = 44 ms). (c) A piece of the wave reenters into the tissue (*t* = 82 ms). (d) New waves are generated and eventually a repeated reentry is formed mimicking a spiral wave (*t* = 122 ms).

**Figure 8:**
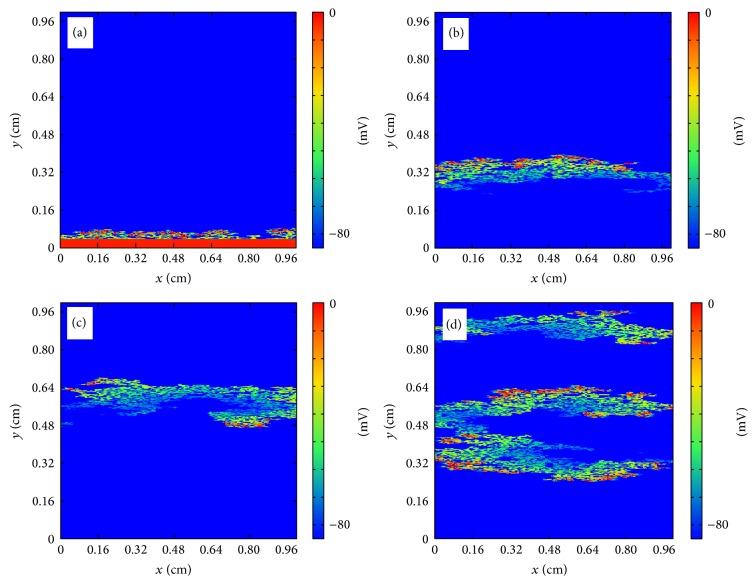
Reentry of action potential generated by the heterogeneous structure of the tissue in the discrete model. (a) Generation of the traveling wave in the bottom part of the tissue (*t* = 10 ms). (b) Wave rapidly propagates to the upper part of the tissue transversely to the fibers direction (*t* = 80 ms). (c) A piece of the wave reenters into the tissue (*t* = 150 ms). (d) New waves are generated and eventually a repeated reentry is formed mimicking a spiral wave (*t* = 220 ms).

In the discrete model, for small fraction *ϕ* propagation is possible and a wave can travel with a reduced velocity from one side of the tissue to the other. For large value of *ϕ* there are no pathways for the wave to propagate and the excitation disappears without the activation of the tissue. For intermediate values of *ϕ* the propagation looks fractionated but it is usually still coherent and travels to the other side of the tissue. However, depending on the random realization of the process of isolation of cells, waves can be broken and it produces the appearance of reentry into the already excited tissue; see Figure 7. A wave of action potential propagates from the left to the right in a tissue with a large fraction of removed cells following the diverse open pathways in the tissue. The propagation is highly fractionated but the wave arrives to the end of the tissue. However, after the left-to-right propagation, a piece of the wave reenters again into the tissue already excited and produces the formation of repeated reentry, equivalent to a spiral wave. The dynamics is stable and the reentry persists for large times, although it could eventually disappear depending on the random realization [24]. Similar patterns of microscopic reentry can be found also by initiating the propagation from the bottom of the tissue; see Figure 8.

We have done a systematic study for the evaluation of the optimal fraction *ϕ* where the probability of reentry is maximal. We run 100 numerical simulations with different random realization for each value of the fraction *ϕ*. The value of *ϕ* was varied from 40% to 55%, in steps of 1%. This allowed us to estimate for each value of *ϕ* the probability of reentry, that is, the ratio of simulations with reentry to the total number of simulations.

**Figure 9:**
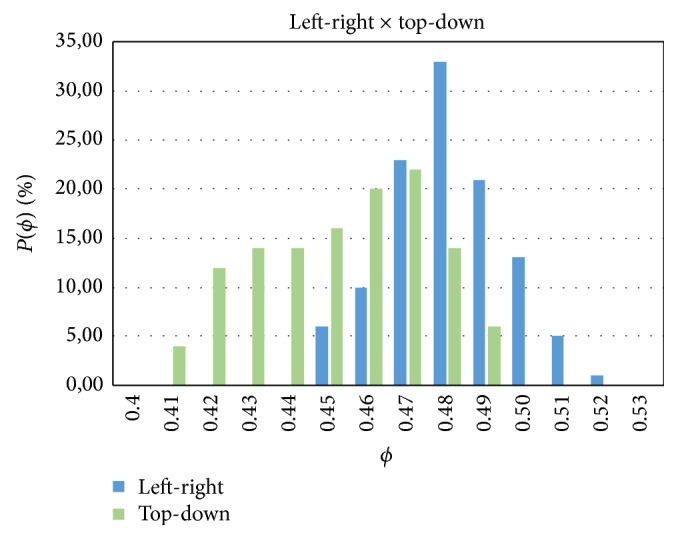
Dependence of the probability of reentry *P*(*ϕ*) of action potential generated by the heterogeneous structure of the tissue in the discrete model on the fraction of nonconnected cells *ϕ*. Comparison between propagation along the fibers (left-right) and perpendicularly to the fibers (top-down).

The results of these simulations are shown in Figure 9. A reentry was identified whenever there was still activity (depolarization) at 120 ms after the initial stimulus for the case of longitudinal propagation, or at 300 ms for the case of transversal propagation. Therefore, here we did not make any difference between sustained or nonsustained reentries. The values of 120 and 300 ms were chosen by checking that a plane wave reaches the end of the tissue for the case of *ϕ* = 0.45 after 60 ms (240 ms) for longitudinal (transverse) propagation. Therefore, in both cases, we checked whether there was still activity 60 ms after the wave was already supposed to be extinguished.

Figure 9 shows a systematic difference of the results depending on the directions of propagation of the waves. In the LP direction the probability of reentry concentrates around *ϕ* = 0.48; however, when waves propagate transversely to the fiber direction (TP) the domain of reentry is broader and the distribution is flatter and above 10% for *ϕ* between 0.42 and 0.48. Nevertheless, in both cases, the probability of reentry goes to zero shortly after *ϕ* ~ 0.5; see Figure 9.

#### 3.2.2. Anatomical Reentry in the Microscopic Model

The numerical simulations using the microscopic model take much longer execution times than those based on the discrete model. The realization of good statistics with such model becomes a very hard task. For this reason, we have chosen the topologies from some selected simulations in the discrete model and implemented them in the microscopic model to compare the results of both models.

We first chose 30 topologies that did not generate reentry in the discrete model, but those were under values of the fraction *ϕ* of high probability of reentry. We observed that none of these simulations using the fine microscopic model gave rise to the appearance of any reentry. Therefore, in this case there was a 100% of match between the two models.

We have also performed 30 (15 TP plus 15 LP simulations) realizations selecting topologies which produced reentry in the discrete model. For the LP case, 15 simulations were executed, five for each value of *ϕ* = {0.45,0.48,0.51}. For the TP case, 15 simulations were also executed, five for each value of *ϕ* = {0.42,0.45,0.48}. Therefore, for both cases we took the value of *ϕ* that maximizes the probability of reentry, and two values of *ϕ* around this value but with low probability of reentry; see Figure 9.

**Figure 10:**
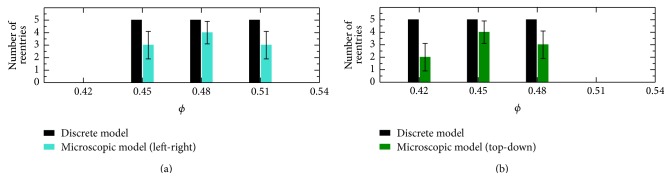
Reentry in the microscopic model in comparison with the discrete model. For each value of *ϕ*, number of simulations that generated reentry into discrete model and also generated in the microscopic model using the same topology, for the left-right (a) and top-down (b) directions.

While 19 realizations out of 30 gave rise to reentry, 11 realizations did not show any reentry. It produces a 63% of agreement between the two models in the prediction of reentries once the reentry appears in the discrete model. These results are shown in Figure 10. The five cases of reentry in the discrete model for each value of *ϕ* reduce to 2–4 reentries in the microscopic model.

**Figure 11:**
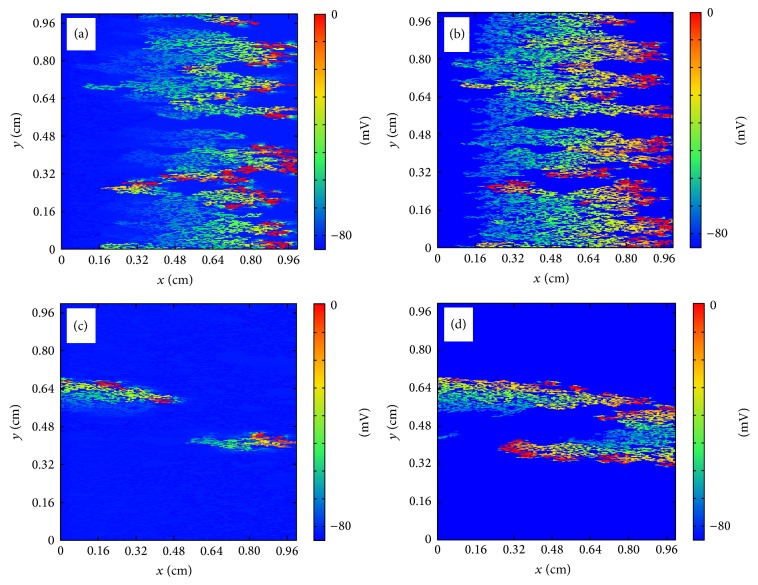
Reentry of action potential generated by the heterogeneous structure of the tissue in the microscopic ((a)-(c)) and the discrete ((b)-(d)) model. ((a)-(b)) Wave rapidly propagates to the right following the direction of the fibers in the microscopic (a) and in the discrete model (b), for *t* = 50 ms. ((c)-(d)) A piece of the wave reenters into the tissue in the microscopic (c) and in the discrete model (d), for *t* = 160 ms.

An example for a case of agreement between the two models is shown in Figure 11. The comparison between the microscopic ((a)-(c)) and the discrete ((b)-(d)) model produces a qualitative agreement in the propagation of the wave and in the localization of the reentry, although the reentrant waves are different in shape.

**Figure 12:**
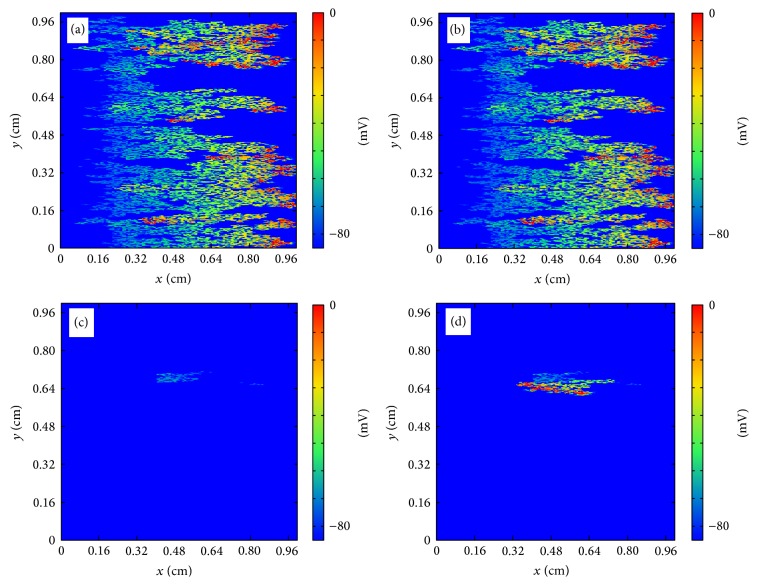
Reentry ((b)-(d)) and nonreentry ((a)-(c)) of action potential generated by the heterogeneous structure of the tissue in the microscopic ((a)-(c)) and the discrete ((b)-(d)) model. ((a)-(b)) Wave rapidly propagates to the right following the direction of the fibers in the microscopic (a) and in the discrete model (b), for *t* = 40 ms. The wave does not reenter into the tissue in the microscopic model (c), for *t* = 100 ms. A piece of the wave reenters into the tissue in the discrete model (d), for *t* = 100 ms.

On the other hand, some realizations using the same topologies for both models gave rise to different final dynamics.  Figure 12 shows the comparison between two simulations with the two models. While we observe a reentry in the discrete model no reentry is observed in the simulation with the microscopic model. The wave initially propagates in the two models following a very similar dynamics; however, later on the simulation, propagation stops in the microscopic model, whereas in the discrete model it persists as a reentry wave.

To further investigate these differences between microscopic and discrete results, we have performed another set of simulations. We have simulated two different fibrotic regions, both with *ϕ* = 0.48, with longitudinal stimulation (left-right) for the duration of 400 ms, and another two different fibrotic regions, both with *ϕ* = 0.45, with transversal stimulation (bottom-up) for the duration of 800 ms. These four new scenarios were simulated with both discrete and microscopic models, that is, a total of 8 simulations. Tables 2 and 3 present the results of this comparison. For each cell (discrete model) or discretized volume (microscopic model) we classified the electrical activity observed during the simulations as NA (no activity), A (a single AP, no reentry), S (sustained reentry, multiple APs until the end of the simulation), or NS (nonsustained reentry, multiple APs, but activity stops, dies out, before the end of the simulation). In addition, for the case of NS activity we have also measured the time the reentry died out.  Table 2 (left-right simulations) and Table 3 (bottom-up simulations) present the percentage of tissue classified as NA, A, S, and NS. We observe a very good match between the results of the discrete and the microscopic models for three out of the four cases studied (both in terms of the classification and the duration of the reentry for the NS case). However, for one particular case (Run 1, Table 2) the discrete model overestimates the area of the tissue that sustains reentry (near one third of the tissue) in comparison to the area where reentry is sustained using the microscopic model (only one fifth of the tissue).

## 4. Discussion

### 4.1. Bridging the Gap between Ectopic Pacemakers Formation and CFAEs in Microfibrosis

Our simulation results present the generation of reentry inside fibrotic tissues. This kind of reentry restricted to microfibrosis regions can result in the formation of ectopic pacemakers, that is, regions that will generate a series of ectopic stimulus at a fast pacing rate. In turn, such activity has been related to trigger fibrillation, both in the atria and in the ventricles in clinical and animal studies [5, 6, 10].

In addition, our results quantitatively described how the percentage of fibrosis (*ϕ*) influences the probability of a region to become an ectopic pacemaker. Reentry activity inside a fibrotic region was identified in cardiac tissues with a percentage of fibrosis (or nonconducting material) between 41% and 52%; see Figure 9. It is interesting to mention that [45] reports that in postmortem atrial tissues from patients who died of cardiovascular causes, the extent of fibrosis was strongly correlated with the history of atrial fibrilation (AF). The mentioned work highlights this correlation presenting three histologies: (1) with a fibrosis extent of 51% of the tissue from a patient with a history of permanent AF; (2) with a fibrosis extent of 14% from a patient with a history of paroxysmal AF; (3) with a fibrosis extent of 5% from a patient with no history of AF. These numbers are in agreement with our findings. Our results suggests that with less than 40% of fibrosis the conduction velocity of the propagating wave can be substantially reduced but without the generation of reentry inside the fibrotic region. This pattern could potentially generate a single ectopic beat, enough for paroxysmal AF. For permanent or sustained AF, the work of [11] suggest the need for an ectopic pacemaker. And indeed the fibrosis extent of 51% reported in [45] and associated with permanent AF is within our predicted values of *ϕ* (between 41% and 52%) that has led to the generation of reentry inside the fibrotic region, that is to the formation of an ectopic pacemaker.

Finally, the computational models used here are very similar to those used in [13] that also simulated microfibrosis with the focus on the study of CFAEs [13]. In [13] it was shown that decoupling of myocytes or increase of fibrosis extent was associated with complex pattern of propagation, discrete propagation, and CFAEs. Since we have studied the generation of ectopic pacemakers with a very similar formalism as used in [13], we have a clear theoretical evidence that brings together ectopic pacemakers and CFAEs for the case of propagation in fibrotic tissues. The results presented here and the new models developed in this work may help in the translational and extremely complex task of relating basic electrophysiological and structural features of cardiac tissue to clinical and relevant procedures such as catheter ablation of atrial fibrillation guided by CFAE mapping [19].

### 4.2. Extending the Percolation Threshold for Anisotropic Cardiac Tissue

**Figure 13:**
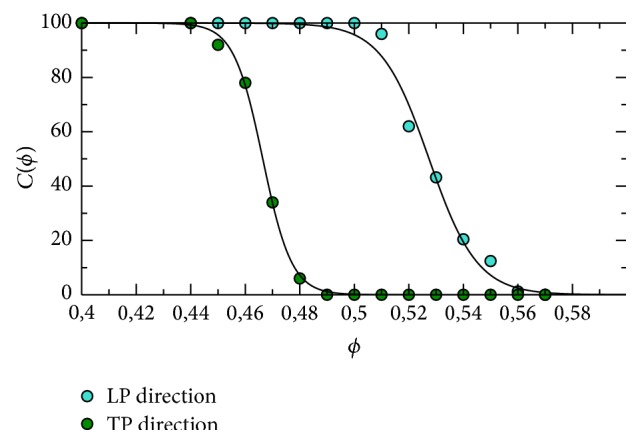
Dependence of the probability of connection between two sides *C*(*ϕ*) in the discrete model on the fraction of nonconnected cells *ϕ*. Comparison between propagation along the fibers (left-right) and perpendicularly to the fibers (top-down). The percolation threshold is determined by the cross of the fitting line with the value *C*(*ϕ*) = 50% with *ϕ*_*c*_ = 0.466 and *ϕ*_*c*_ = 0.527 for TP and LP directions, respectively.

The maximum in the probability of reentry has been previously associated with the percolation threshold of the network or topology of the tissue [24]. Here, as already mentioned, the average number of first neighbors of the network (see Figure 3(b)) is around 6 and the topological structure of the cell network is similar to an hexagonal network, whose percolation threshold is *ϕ* = 0.5. However, the network used here is not exactly hexagonal and represents an anisotropic tissue. The anisotropic conduction does not only elongates the symmetric view in one direction but also modifies the distribution of the first neighbors. As a result, the percolation threshold changes. In addition, as suggested by the results of Figure 9, it is likely that the percolation threshold not only depends on the topology of the network or tissue but also on the direction of the propagating wave. To verify this hypothesis, we have numerically calculated the probability of connection between two opposing sides of the tissue, *C*(*ϕ*), using the same method as proposed in [24], for the two different propagating directions, TP and LP.  Figure 13 presents the results for the probability of connection, *C*(*ϕ*), for the two cases, TP and LP. From these two distributions we can obtain that in the LP direction the percolation threshold is *ϕ* = 0.527, whereas for the TP direction it is *ϕ* = 0.47.

Therefore, we conclude that the percolation threshold for anisotropic cardiac tissue depends not only on the topology of the connection between the myocytes but also on the direction of propagation with respect to the cardiac fiber orientation. Nevertheless, as presented in Figure 9, the range of values for percolation threshold, a pure topological or anatomical metric, is still very important. Even in the case of tissue anisotropy, this range of values for percolation threshold (0.47–0.52) marked the percentage of fibrotic tissue, *ϕ*, needed for a tissue to have a high probability to generate reentry and become an ectopic pacemaker.

**Figure 14:**
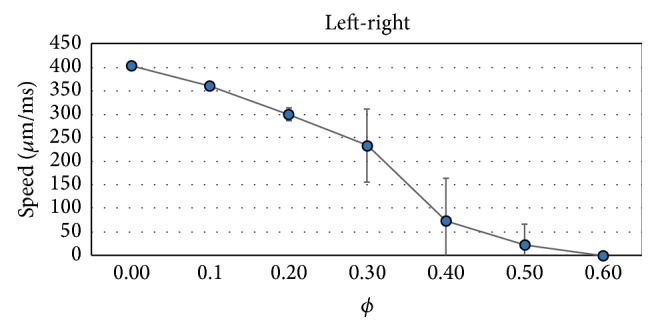
Dependence of the conduction velocity on the fraction of nonconnected cells *ϕ* for the propagation along the fibers (left-right). Average and STD values.

**Figure 15:**
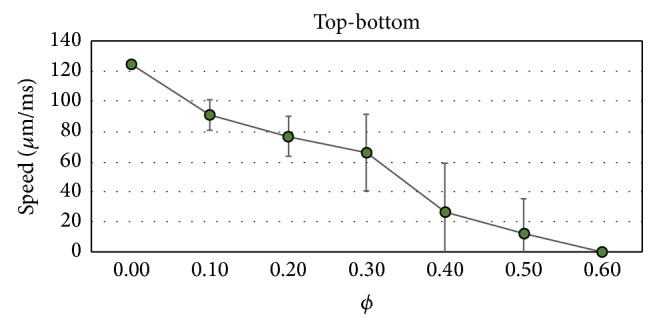
Dependence of the conduction velocity on the fraction of nonconnected cells *ϕ* for the propagation transversal to the fibers (bottom-up). Average and STD values.

In addition, similar to Figure 9, Figures 14 and 15 present how the conduction velocity (CV) depends on *ϕ* for the case of LP and TP propagation directions, respectively. The figures present the results of average and STD of the calculated values of CV. We can observe the existence of extremely slow propagating waves for *ϕ* near the percolation threshold.

### 4.3. A New Discrete Model for Cardiac Tissue

We developed and tested a new discrete model which is a reduced and simplified version of a complex microscopic model. To obtain the same physiological conditions, we carefully tuned the parameters of the connectivities. As a result of the fitting, traveling and spiral waves propagate with very similar pattern and velocities in both models, discrete and microscopic, under homogeneous conditions; that is, we have observed a quantitative match of the two models. Therefore, we can conclude that the new developed discrete model is a good alternative for the microscopic one under homogeneous conditions, since it offers simulations that are much faster, in terms of execution times, than those using the microscopic model.

We have also compared the microscopic and the discrete models under heterogeneous conditions. Both models have shown the same qualitative behavior when the probability of reentry was studied on fibrotic tissues. However, quantitative differences were also clearly present. Out of the 60 simulations that we have chosen to compare the two models, in 49 the two models matched (in terms of reentry generation), that is, 82% of agreement. However, within the cases where reentries were observed using the discrete model, 30 cases, only 19 also generated reentry when using the microscopic model, which drops the matches to 63%.

**Figure 16:**
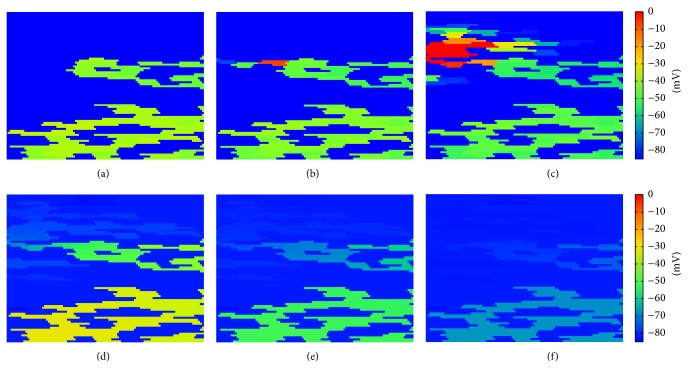
Reentry in the microscopic model in comparison with the discrete model. Panels (a) (*t* = 75 ms), (b) (*t* = 80 ms), and (c) (*t* = 90 ms), successful reentry propagation (from right to left of the tissue) using the discrete model. Panels (d) (*t* = 75 ms), (e) (*t* = 80 ms), and (f) (*t* = 90 ms), reentry dies out using the microscopic model due to source-sink mismatch.

Therefore, our preliminary results suggest that the discrete model overestimates the probability of reentry, if we take the microscopic model as our gold standard. We have taken a close look at the simulations where the two models did not match.  Figure 16 presents an example of these comparisons. In panels (a), (b), and (c), we observe a successful reentry propagation (from right to left in the tissue) using the discrete model. In panels (d), (e), and (f), we observe the corresponding situation using the microscopic model, where reentry dies out. Comparing panels (a) and (d) we observe that current spreads out more easily in the microscopic model (panel (d)) than in the discrete model (panel (a)). This is due to the continuous nature of the microscopic model. As a result, current sink is larger in the microscopic model than in the discrete model. This classical source-sink mismatch [46] blocks the propagation of the reentry in the microscopic model. On the other hand, the lumped nature of the discrete model seems to help propagation by reducing the sink area.

Nevertheless, we reinforce that, even in these cases of microfibrosis simulation, we can claim that the results of the discrete model matched qualitatively those of the microscopic model, with accuracy between 60% and 80%. Since this study involved the simulations of more than three thousands of different fibrotic tissues, we note that these statistics would not be possible to generate in a timely fashion without the aid of the new discrete model.

### 4.4. Limitations and Future Work

All the simulations shown in this study were carried out in a completely damaged area. Fibrosis is typically localized in the heart and the damaged area coexists with healthy surrounding. In [24], two different simulations were performed: one considered a completely damaged area, that is, a fibrotic region as performed here in this work, and another larger setup considered a fibrotic region surrounded by healthy tissue. In [24] it was shown that most of the reentries generated inside the fibrotic region, but not all of them, could propagate to the healthy tissue. In future studies we will use the same methods presented here to simulate the setup of a fibrotic region surrounded by healthy tissue and quantify, as performed here, how features of fibrosis are related to the generation of ectopic pacemakers.

We have fixed the size of the simulation area to 1 × 1 cm^2^. However, the probability to find an ectopic beat is proportional to the probability to have locally the adequate conditions for a source-link mismatch [46]. Such probability depends on the size of the system, the larger the system, the higher the probability to obtain an ectopic beat. In future studies on a systematic variation of the domain of integration will help to identify a minimum size for the generation of reentry.

Two different dynamics have been previously obtained in [24], sustained and nonsustained reentries. The last case produce a single ectopic beat without any repetition and with a mild effect in the cardiac muscle. The first one is associates with ectopic pacemakers. In this work, we did not quantitatively differentiate between sustained and nonsustained reentries. In future works, longer simulations will be performed to better classify how many of the simulated reentries are sustained and how many are not.

We have employed the Bondarenko model [35] for mouse ventricle. A natural next step in terms of translational science is to replace this model with a human atrial or ventricular model. In addition, other important electrophysiological changes should be taken into account by the models in order to simulate fibrosis. In future works, a more complete description of fibrosis may include, for example, gap junction remodeling as well as myocyte-fibroblast coupling [47].

Finally, we want to note that we considered two dimensional slabs of tissue. It is usually a strong simplification of the geometry of the tissue. In the near future we will consider three-dimensional simulations. In this case, the introduction of an additional dimension will probably change the topological conditions of the grid (percolation threshold will change from 0.5% to larger values) but not the phenomenology around the percolation threshold as it was anticipated in [24].

## 5. Conclusions

We developed and tested a new discrete model which is a reduced and simplified version of a detailed subcellular microscopic model that accounts for the microstructure of the cardiac tissue including distribution of cells and gap junctions. Our preliminary results suggest a very good agreement between the simulations of the new discrete model and those obtained by the detailed and subcellular microscopic model. The new discrete model was used for the simulation of fibrotic regions. Our results quantitatively described how the percentage of fibrosis (*ϕ*) influences the probability of a region to become an ectopic pacemaker. Reentry activity inside a fibrotic region was identified in cardiac tissues with a percentage of fibrosis (or nonconducting material) between 41% and 52%. In addition, our results suggest that the probability of reentry inside a fibrotic region not only depends on *ϕ* but also on the direction of propagation with respect to the cardiac fiber orientation; that is, tissue anisotropy also plays an important role.

In summary, our simulation results present the generation of reentry inside fibrotic tissues. Therefore, we were able to generate an ectopic pacemaker in computer simulations of cardiac electrophysiology by assuming a single microstructural or anatomical hypothesis for substrate: the existence of a mixture of excitable and nonexcitable regions. The two different phases (excitable and nonexcitable) form a maze or labyrinth for wave propagation. Propagation becomes fractioned and the topology of the maze allows the electric wave to reexcite the fibrotic tissue before the wave leaves it. Our results can be combined with previous works that used a very similar computational model to study CFAEs. Therefore, we present a clear theoretical evidence that brings together ectopic pacemakers and complex fractionated electrograms for the case of propagation in fibrotic tissues that may become triggers of fibrillation.

## Figures and Tables

**Table 1:** Average of execution times (in seconds) for simulations of a 1.0 cm × 1.0 cm tissue for 10 ms. Each case (line of table) was run three times.

Implementation model	Computing cores	Total execution time (s)
Sequential-microscopic	1	546507
Multi-GPU-microscopic	64 + 16 GPUs	1302
Multi-GPU-discrete	64 + 16 GPUs	65

**Table 2:** Left-right, ϕ = 0.48. Each cell (discrete model) or discretized volume (microscopic model) was classified as NA (no activity), A (a single AP, no reentry), S (sustained reentry, multiple APs until the end of the simulation), or NS (nonsustained reentry, multiple APs, but activity stops, dies out, before the end of the simulation). The table presents the percentage of area of the tissue for each category. In addition, for the category NS we present the time the reentry dies out or stops.

Type	Run 1		Run 2	
	Microscopic	Discrete	Microscopic	Discrete
NA	54.1%	51.7%	55%	53.5%
OA	18.3%	16.2%	15.6%	14.4%
S	19.2%	32.1%	17.3%	20.9%
NS	8.4% (stops at 216 ms)	0%	12.1% (stops at 223 ms)	11.2% (stops at 217 ms)

**Table 3:** Top-down, ϕ = 0.45. Each cell (discrete model) or discretized volume (microscopic model) was classified as NA (no activity), A (a single AP, no reentry), S (sustained reentry, multiple APs until the end of the simulation), or NS (nonsustained reentry, multiple APs, but activity stops, dies out, before the end of the simulation). The table presents the percentage of area of the tissue for each category. In addition, for the category NS we present the time the reentry dies out or stops.

Type	Run 3		Run 4	
	Microscopic	Discrete	Microscopic	Discrete
NA	60.9%	59.8%	62.7%	61.1%
OA	14.3%	13.8%	21.2%	20.6%
S	17.6%	19.9%	7.1%	9.9%
NS	7.2% (stops at 391 ms)	6.5% (stops at 389 ms)	9% (stops at 368 ms)	8.4% (stops at 365 ms)
